# Comparative efficacy of selenoureido carbonic anhydrase inhibitors and azole antifungal drugs against clinical isolates of *Malassezia pachydermatis*

**DOI:** 10.1111/vde.13336

**Published:** 2025-03-16

**Authors:** Costanza Spadini, Nicolò Mezzasalma, Amienwanlen Eugene Odigie, Andrea Angeli, Fabrizio Carta, Silvia Selleri, Emanuele Gandolfo, Simone Taddei, Valentina Franceschi, Sergio Minesso, Claudiu T. Supuran, Clotilde Silvia Cabassi

**Affiliations:** ^1^ Department of Veterinary Science University of Parma Parma Italy; ^2^ Department of Veterinary Medicine University of Bari “Aldo Moro” Bari Italy; ^3^ Section of Pharmaceutical and Nutraceutical Science, NEUROFARBA Department University of Florence Florence Italy; ^4^ Veterinary Practitioner Veterinary Center “Giuseppe Verdi” Parma Italy

**Keywords:** azoles, carbonic anhydrases inhibitors, dermatomycosis, dogs, Malassezia, selenium

## Abstract

**Background:**

*Malassezia pachydermatis* (MP) is implicated in severe dermatitis and otitis externa (OE) of companion animals and recently gained attention for its increasing resistance to azole compounds. For this reason, developing novel therapeutic strategies is of great interest. In a previous work, we used reference yeast isolates to evaluate several compounds bearing acyl/selenoureido moieties and primary/secondary sulfonamide groups for antifungal activity through organic selenium and carbonic anhydrase inhibition.

**Objectives:**

This work aimed to evaluate the antifungal efficacy of eight selenoureido compounds on 36 clinical MP isolates from dogs, compared to selected azoles, notably ketoconazole (KCZ), miconazole (MCZ) and fluconazole (FCZ).

**Materials and Methods:**

MIC assays of **5g**, **7a**, **7c**, **7k**, **8c**, **10c**, **11b**, **11f**, KCZ, MCZ and FCZ were performed on 36 MP field isolates isolated from dogs affected by dermatitis and/or OE in which yeast aetiology was suspected. Minimum 50% and 90% inhibitory concentrations (MIC_50_ and MIC_90_) were calculated. MP identification was confirmed with a nested PCR for the internal transcribed spacer region of the rRNA gene.

**Results:**

Overall, the MIC_50_ of the tested compounds on MP field isolates was higher than the MICs obtained on reference MP DSM 6172. Although KCZ showed the lowest MIC_50_ value, compounds **5g**, **7a** and **7k** showed lower MIC_50_s than MCZ and FCZ. Five clinical isolates showed a MIC on azoles >MIC_90_. Compounds **7a** (four of five), **10c** (three of five) and **8c** (three of five) showed lower MIC values on these isolates compared to the tested azoles, suggesting good activity in phenotypically azole‐resistant MP.

**Conclusions and Clinical Relevance:**

Considering the increasing azole resistance of the *Malassezia* genus, selenoureido compounds could represent a potential topical treatment for dog skin and ear mycotic infections.

## Graphical abstract

**Background** – *Malassezia pachydermatis* (MP) is implicated in severe dermatitis and otitis externa (OE) of companion animals and recently gained attention for its increasing resistance to azole compounds. For this reason, developing novel therapeutic strategies is of great interest. In a previous work, we used reference yeast isolates to evaluate several compounds bearing acyl/selenoureido moieties and primary/secondary sulfonamide groups for antifungal activity through organic selenium and carbonic anhydrase inhibition. **Objectives** – This work aimed to evaluate the antifungal efficacy of eight selenoureido compounds on 36 clinical MP isolates from dogs, compared to selected azoles, notably ketoconazole (KCZ), miconazole (MCZ) and fluconazole (FCZ). **Conclusions and Clinical Relevance** – Considering the increasing azole resistance of the *Malassezia* genus, selenoureido compounds could represent a potential topical treatment for dog skin and ear mycotic infections.

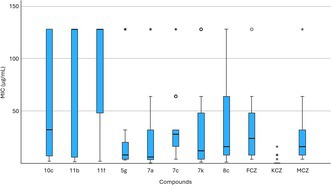

## Zusammenfassung

**Hintergrund:**

*Malassezia pachydermatis* (MP) ist bei schweren Dermatitiden und Otitis externa (OE) von Haustieren beteiligt und hat jüngst wegen seiner zunehmenden Resistenz gegen Azol Bestandteile Aufmerksamkeit erregt. Aus diesem Grund ist die Entwicklung neuer therapeutischer Strategien von größtem Interesse. In einer früheren Studie verwendeten wir Referenz Hefepilzstamm Isolate, um einige Bestandteile, wie Acyl/Selenoureidomoiëten und primäre/sekundäre Sulfonamidgruppen in Bezug auf ihre anti‐mykotische Wirkung durch organisches Selen und Carboanhydrasen Inhibition zu evaluieren.

**Ziele:**

Diese Arbeit zielte darauf ab, die antimykotische Wirksamkeit von acht Selenoureido‐ Mischungen auf 36 klinische MP Isolate von Hunden, im Vergleich zu ausgewählten Azolen, vor allem Ketokonazol (KCZ), Miconazol (MCZ) und Fluconazol (FCZ) zu evaluieren.

**Materialien und Methoden:**

MIC Assays von 5g, 7a, 7c, 7k, 8c, 10c, 11b, 11f, KCZ, MCZ und FCZ wurden mit 36 MP Feldisolaten, die von Hunden mit einer Dermatitis und/oder OE bei denen Hefen als Ursache vermutet wurden, durchgeführt. Es wurde die 50%ige und 90%ige minimale Hemmkonzentration (MIC_50_ und MIC_90_) kalkuliert. Die MP Identifizierung wurde mit einem Nested PCR für die Internal transcribed Spacer Region des rRNA‐Gens bestätigt.

**Ergebnisse:**

Insgesamt war die MIC_50_ der getesteten Mischungen auf die MP Feldisolate höher als die MICs der Referenz MP DSM 6172. Obwohl KCZ die niedrigsten MIC_50_ Werte zeigte, wiesen die Mischungen 5g, 7a und 7k niedrigere MIC_50_ auf als MCZ und FCZ. Fünf klinische Isolate zeigten eine MIC für Azole von >MIC_90_. Mischung 7a (vier und fünf), 10c (drei und fünf) und 8c (drei und fünf) zeigten niedrigere MIC‐Werte für diese Isolate im Vergleich zu den getesteten Azolen, was auf eine gute Aktivität bei phänotypisch Azol‐resistenten MP hinweist.

**Schlussfolgerungen und klinische Bedeutung:**

Wenn man die zunehmende Azol‐Resistenz von Malassezia Stämmen bedenkt, könnten Selenoureido‐Mischungen eine mögliche topische Behandlung von Hundehaut und Pilzinfektion des Ohres darstellen.

## 摘要

**背景:**

厚皮马拉色菌 (MP) 与伴侣动物的严重皮炎和外耳炎 (OE) 有关，最近因其对唑类化合物的耐药性不断增加而受到关注。因此，开发新的治疗策略具有重要意义。在之前的研究中，我们使用标准酵母菌分离株评估几种含有酰基/硒脲基部分和一级/二级磺酰胺基团的化合物，其通过有机硒和碳酸酐酶抑制发挥抗真菌活性。

**目标:**

这项研究旨在评估八种硒脲基化合物对 36 种临床犬 MP 分离株的抗真菌效果，并与选定的唑类药物(特别是酮康唑 (KCZ)、咪康唑 (MCZ) 和氟康唑 (FCZ))进行比较。

**材料与方法:**

对疑似由酵母菌引起的皮炎和/或外耳炎(OE)患犬身上分离出的36个MP田野分离株，进行了5g、7a、7c、7k、8c、10c、11b、11f、KCZ、MCZ和FCZ的MIC(最低抑制浓度)试验。计算了最低50%和90%抑制浓度(MIC50和MIC90)。通过针对rRNA基因内部转录间隔区的巢式PCR来确认MP的鉴定。

**结果:**

总体而言，测试化合物对 MP 田野分离株的 MIC50 高于对参考 MP DSM 6172 获得的 MIC。虽然 KCZ 显示出最低的MIC50值，但化合物5g、7a和7k 显示出比 MCZ 和 FCZ 更低的 MIC50。五种临床分离株对唑类的 MIC>MIC90。与测试的唑类相比，化合物 7a(五种中的四种)、10c(五种中的三种)和 8c(五种中的三种)对这些分离物表现出较低的 MIC 值，表明在表型上唑类耐药的 MP中具有良好的活性。

**结论和临床意义:**

考虑到马拉色菌属的唑类耐药性不断增加，硒脲类化合物可能代表一种治疗犬皮肤和耳朵真菌感染的潜在局部药物。

## Résumé

**Contexte:**

*Malassezia pachydermatis* (MP) est impliqué dans les dermatites sévères et les otites externes (OE) des animaux de compagnie et a récemment attiré l'attention sur sa résistance croissante aux composés azolés. C'est pourquoi le développement de nouvelles stratégies thérapeutiques est d'un grand intérêt. Dans un travail précédent, nous avons utilisé des isolats de levure de référence pour évaluer l'activité antifongique de plusieurs composés portant des motifs acyl/sélénoureido et des groupes sulfonamides primaires/secondaires par le biais de l'inhibition du sélénium organique et de l'anhydrase carbonique.

**Objectifs:**

Ce travail visait à évaluer l'efficacité antifongique de huit composés de sélénoureido sur 36 isolats cliniques de MP provenant de chiens, en comparaison avec des azoles sélectionnés, notamment le kétoconazole (KCZ), le miconazole (MCZ) et le fluconazole (FCZ).

**Matériels et méthodes:**

Les CMI de 5g, 7a, 7c, 7k, 8c, 10c, 11b, 11f, KCZ, MCZ et FCZ ont été réalisées sur 36 isolats de terrain de MP isolés de chiens atteints de dermatite et/ou d'OE pour lesquels une étiologie levurienne était suspectée. Les concentrations minimales inhibitrices à 50 % et 90 % (CMI_50_ et CMI_90_) ont été calculées. L'identification des MP a été confirmée par une PCR nichée pour la région de l'espace transcrit interne du gène de l'ARNr.

**Résultats:**

Dans l'ensemble, la CMI_50_ des composés testés sur les isolats de terrain de MP était plus élevée que les CMI obtenues sur le MP de référence DSM 6172. Bien que le KCZ ait montré la valeur de CMI_(50) la_ plus basse, les composés 5g, 7a et 7k ont montré des CMI_50_ inférieures à celles du MCZ et du FCZ. Cinq isolats cliniques ont présenté une CMI aux azoles >CMI_90_. Les composés 7a (quatre sur cinq), 10c (trois sur cinq) et 8c (trois sur cinq) ont montré des valeurs de CMI inférieures sur ces isolats par rapport aux azoles testés, ce qui suggère une bonne activité sur les MP phénotypiquement résistants aux azoles.

**Conclusions et pertinence clinique:**

Compte tenu de la résistance croissante du genre *Malassezia* aux azoles, les composés selenoureido pourraient représenter un traitement topique potentiel des infections mycosiques de la peau et de l'oreille du chien.

## 要約

**背景:**

*Malassezia pachydermatis*:MPは、コンパニオンアニマルの重篤な皮膚炎や外耳炎(OE)に関与しており、近年ではアゾール系化合物に対する耐性が増加していることで注目されている。このため、新たな治療戦略の開発が大きな関心を集めている。以前の研究では、参照酵母分離株を用いて、acyl/selenoureido moietiesおよび1級/2級スルホンアミド基を有するいくつかの化合物について、有機セレン阻害および炭酸脱水酵素阻害による抗真菌活性を評価した。

**目的:**

本研究の目的は、イヌの臨床 MP 分離株 36 株に対する 8 種類のセレノウレイド化合物の抗真菌効果を、特定のアゾール系抗真菌薬、特にケトコナゾール(KCZ)、ミコナゾール(MCZ)およびフルコナゾール(FCZ)と比較検討することであった。

**材料と方法:**

5g、7a、7c、7k、8c、10c、11b、11f、KCZ、MCZおよびFCZのMIC測定を、酵母の病因が疑われる皮膚炎および/またはOEに罹患したイヌから分離された36のMP野外分離株に対して行った。最小50%および90%阻害濃度(MIC_50_およびMIC_90_)を算出した。MPの同定はrRNA遺伝子の内部転写スペーサー領域に対するnested PCRで確認した。

**結果:**

全体的に、試験化合物のMP野外分離株に対するMIC_50_は、基準MP DSM 6172で得られたMICよりも高かった。KCZが最も低いMIC_50_値を示したが、化合物5g、7aおよび7kはMCZおよびFCZよりも低いMIC_50_値を示した。臨床分離株5株はアゾール系抗菌薬に対するMICがMIC_90_以上であった。化合物7a(5株中4株)、10c(5株中3株)および8c(5株中3株)は、これらの分離株に対して、試験したアゾール系薬剤と比較して低いMIC値を示し、表現型的にアゾール耐性のMPに良好な活性を示すことが示唆された。

**結論と臨床的意義:**

マラセチア属のアゾール耐性の増加を考慮すると、セレノウレイド化合物は犬の皮膚および耳の真菌感染症の局所治療薬となる可能性があった。

## Resumo

**Contexto:**

*Malassezia pachydermatis* (MP) está envolvida em dermatite e otite externa (OE) graves em animais de estimação e recentemente ganhou atenção por sua crescente resistência a compostos azólicos. Por esse motivo, desenvolver novas estratégias terapêuticas é muito importante. Em um trabalho anterior, utilizamos isolados de levedura de referência para avaliar a atividade antifúngica de diversos compostos contendo frações acil/selenoureído e grupos sulfonamida primários/secundários por meio da inibição do selênio orgânico e da anidrase carbônica.

**Objetivos:**

Este estudo teve como objetivo avaliar a eficácia antifúngica de oito compostos de selenoureído em 36 isolados clínicos de MP de cães, em comparação dos seguintes azóis selecionados: cetoconazol (KCZ), miconazol (MCZ) e fluconazol (FCZ).

**Materiais e métodos:**

Ensaios de MIC de 5g, 7a, 7c, 7k, 8c, 10c, 11b, 11f, KCZ, MCZ e FCZ foram realizados em 36 isolados de campo de MP isolados de cães afetados por dermatite e/ou OE nos quais havia suspeita de etiologia de levedura. Foram calculadas concentrações inibitórias mínimas de 50% e 90% (CIM50 e CIM90). A identificação da MP foi confirmada com uma PCR aninhada para a região espaçadora transcrita interna do gene rRNA.

**Resultados:**

No geral, o MIC50 dos compostos testados em isolados de MP de foram maiores do que os MICs obtidos para MP de referência DSM 6172. Embora KCZ tenha apresentado o menor valor de MIC50, os compostos 5g, 7a e 7k apresentaram MIC50s menores do que MCZ e FCZ. Cinco isolados clínicos apresentaram MIC em azóis >MIC90. Os compostos 7a (quatro de cinco), 10c (três de cinco) e 8c (três de cinco) apresentaram valores de MIC mais baixos nesses isolados em comparação aos azóis testados, sugerindo boa atividade em MP fenotipicamente resistentes a azóis.

**Conclusões e relevância clínica:**

Considerando a crescente resistência do gênero *Malassezia* aos azóis, os compostos de selenoureÍdo podem representar um potencial tratamento tópico para infecções micóticas de pele e orelhas em cães.

## RESUMEN

**Introducción:**

*Malassezia pachydermatis* (MP) está implicada en la dermatitis severa y en otitis externa (EO) de los animales de compañía y recientemente ha ganado atención por su creciente resistencia a los compuestos azólicos. Por esta razón, el desarrollo de nuevas estrategias terapéuticas es de gran interés. En un trabajo anterior, utilizamos aislados de levadura de referencia para evaluar varios compuestos que contienen grupos acilo/selenoureido y grupos sulfonamida primarios/secundarios para la actividad antifúngica a través de la inhibición de la anhidrasa carbónica y el selenio orgánico.

**Objetivos:**

Este trabajo tuvo como objetivo evaluar la eficacia antifúngica de ocho compuestos selenoureido en 36 aislados clínicos de MP de perros, en comparación con azoles seleccionados, en particular ketoconazol (KCZ), miconazol (MCZ) y fluconazol (FCZ).

**Materiales y métodos:**

Se realizaron ensayos de MIC de 5g, 7a, 7c, 7k, 8c, 10c, 11b, 11f, KCZ, MCZ y FCZ en 36 aislamientos de campo de MP aislados de perros afectados por dermatitis y/o EO en los que se sospechó etiología por levaduras. Se calcularon concentraciones inhibitorias mínimas del 50% y del 90% (MIC_50_ y MIC_90_). La identificación de MP se confirmó con una PCR en nido para la región espaciadora transcrita interna del gen RNAr.

**Resultados:**

En general, la MIC_50_ de los compuestos evaluados en los aislamientos de campo de MP fue mayor que las MIC obtenidas en la referencia MP DSM 6172. Aunque KCZ mostró el valor de MIC_50_ más bajo, los compuestos 5g, 7a y 7k mostraron MIC_50_ más bajas que MCZ y FCZ. Cinco aislamientos clínicos mostraron una MIC en azoles > MIC_90_. Los compuestos 7a (cuatro de cinco), 10c (tres de cinco) y 8c (tres de cinco) mostraron valores de MIC más bajos en estos aislados en comparación con los azoles evaluados, lo que sugiere una buena actividad en MP fenotípicamente resistente a los azoles.

**Conclusiones y relevancia clínica:**

Considerando la creciente resistencia a los azoles del género *Malassezia*, los compuestos selenoureido podrían representar un posible tratamiento tópico de las infecciones micóticas de la piel y los oídos de los perros.

## INTRODUCTION

Yeasts of the genus *Malassezia* are skin commensals of mammals which lack fatty acid synthetase genes. This condition is related to their strong lipid‐dependent metabolism, which contributes to the close bond with mucosae and skin surfaces of the hosts.^1^ The lipid dependence of the *Malassezia* genus is linked to the high expression of lipase and phospholipase genes, which also are implicated in the induction of pro‐inflammatory genes on keratinocytes.^2^ Currently, *Malassezia* comprises 18 species, of which *M. pachydermatis* (MP) is the most represented in the cutaneous mycobiome of animals, both as a commensal and a pathogen, while in humans several species are reported.^3^ Although historically MP was regarded as only lipophilic, owing to its ability to grow on Sabouraud's dextrose agar (SDA), its recent genome sequencing confirmed a lipid dependency, with a peculiar ability to utilise lipid fractions of peptone within the medium.^4^, ^5^ Predisposing factors for MP overgrowth on dog skin (breed, climate, cutaneous hypersensitivity disorders or primary/secondary seborrhoeic dermatoses) are related to the onset of inflammatory diseases causing otitis externa (OE) and/or dermatitis.^6^ The clinical presentation of MP dermatitis can vary, yet erythema with kerato‐sebaceous scales in intertriginous areas is the most frequently detected, while OE often begins with pruritus and erythema followed by hyperkeratosis and lichenification of pinnae and external ear canals in chronic cases.^1^

Therapeutic protocols for MP infections in companion animals involve both topical and systemic antifungals, among which azole derivatives are the most used (miconazole [MCZ], ketoconazole [KCZ], itraconazole, fluconazole [FCZ]), followed by terbinafine and, rarely and only for topical treatment, chlorhexidine and selenium disulfide (SeS_2_).^7^ As indicated by the consensus guidelines, for MP dermatitis (MD) shampoos with 2% miconazole and 2% chlorhexidine twice weekly should be the treatment of first choice.^8^, ^9^ For MP otitis (MO) oral treatment is recommended generally only in case of recurrence, as the topical treatment is in most cases effective.^10^

Antifungal treatments for MD and MO are effective to control MP overgrowth when primary causes and predisposing factors are resolved first; however, a possible cause of therapeutic failure may be the onset of antifungal resistance.^10^

In human medicine, antifungal resistance has been progressively increasing, particularly after the COVID‐19 pandemic, leading the World Health Organization (WHO) to draft the Fungal Priority Pathogens List (FPPL) of public health importance, with the aim to prioritizUpone fungal pathogens and promote research against invasive fungal diseases.^11^ Although the FPPL does not include MP, as it is not responsible for fatal mycoses such as the other pathogens included in the list, this yeast could represent a public concern, as a consequence of its zoonotic potential and its reported resistance to azoles antifungals.^1^, ^12^, ^13^, ^14^

Considering the topical administration of inorganic SeS_2_—which acts on the sterol pathways by means of multiple and yet to be revealed mechanisms—the effectiveness of selenium‐based drugs is closely related to the uptake capacity of the organism, which in turn is highly dependent on its lipidome constitution.^15^, ^16^ In a previous work, Carta et al. investigated the organic form of selenium within scaffolds showing great antifungal effectiveness and selectivity against reference isolates of MP, *M. furfur* (MF) and *M. globosa* (MG).^17^ Tested compounds were endowed with organic scaffolds carrying primary/secondary sulfonamide moieties to target the fungal‐expressed metalloenzyme carbonic anhydrases (CAs, E.C. 4.2.1.1). Such enzymes, among others, have shown properties as novel drug targets because they regulate key metabolic transformations related to fungal survival and virulence.^18^, ^19^, ^20^, ^21^

The present work aimed to evaluate eight selenoureido compounds as antifungals against a collection of 36 field isolates of MP isolated from dogs affected by dermatitis and/or OE. These compounds are part of a larger library previously tested by this research group against reference isolates of MP, MF, MG, *Candida albicans* and *C. glabrata*.^14^ As comparisons for the efficacy of these selenoureido compounds, three azole antifungals were considered (KCZ, MCZ and FCZ).

## MATERIALS AND METHODS

### Sample collection

Fifty samples were obtained from ear (n = 35) or cutaneous (*n* = 15) swabs collected from dogs presented to the veterinary practitioner. Patients were admitted to the Veterinary Teaching Hospital of the Department of Veterinary Science (University of Parma) and to the Veterinary Center “Giuseppe Verdi” (Traversetolo. Parma, Italy), both located in Parma province. Patients were affected by OE and/or dermatitis, with clinical signs that were consistent with a *Malassezia* spp. infection, namely pruritic dermatitis/otitis with moderate to severe erythematous and/or seborrhoeic lesions accompanied by self‐trauma and malodor. During the specialist examination, a cytological test using modified Wright–Giemsa staining (Diff‐Quik; Harleco) was performed on patients with a suspected yeast aetiology. A tape‐strip impression test was performed on skin lesions compatible with MP infection. Lesions with typical *Malassezia* cells visible under the light microscope were then sampled with a sterile cotton‐tipped swab and sent to the laboratory in Amies transport medium within 12 h of sampling, maintaining a cool temperature during transport.

Dogs were sampled both before and after antifungal treatment to assess the persistence of *Malassezia* cells. Treatment was prescribed by the practitioner for ≥20 days and adapted on a case‐by‐case basis based on the patient's condition. The type and duration of treatment were recorded.

### Isolation of *M. pachydermatis*

Upon receipt at the laboratory, swabs were promptly plated onto SDA (Difco) and incubated at 37°C in aerobic conditions for 48 h. After incubation, a first yeast identification was based on colony growth and characteristics; then, on suspected yeast colonies, a cell staining with methylene blue was performed, evaluating the typical morphology of MP cells. Positive cultures were isolated and amplified onto CHROM‐agar *Malassezia* (CHROM‐agar) agar plates. Yeast colonies with a mucoid pink‐to‐purple appearance were suspected as MP and re‐plated onto SDA and incubated at 37°C for 48 h. Subsequently, the colonies were subjected to DNA extraction and a nested PCR was performed for MP identification.

For DNA extraction, single colonies from pure cultures on SDA were selected, and the MasterPure Yeast DNA Purification Kit MPY80200 (Epicentre Technologies) was applied according to the manufacturer's instructions. Finally, DNA was suspended in Tris‐EDTA (TE) buffer and stored at −20°C.

Nested PCR was performed as described by Sugita et al.^22^ using two sets of primers derived from the internal transcribed spacer region 1 (ITS1) of the rRNA gene. The first set of primers specific for the *Malassezia* genus was ITS1F‐N: 5′➔3′ GGATCATTAGTGATTGCCTTTATA and ITS4‐R: 3′➔5′ TCCTCCGCTTATTGATATG. The second set of primers specific for *M. pachydermatis* was M.pa‐F: 5′➔3′ CTGCCATACGGATGCGCAAG and 5.8S‐R: 3′➔5′ TTCGCTGCGTTCTTCATCGA (220 bp). Extracted DNA (5 μL) from each sample was added to 20 μL of PCR mixture comprising: 5 μL of PCR buffer (5×); 5 μL of MgCl_2_ 25 mm, 2.5 μL of the first set of primers 10× (2.5 μM each primer), 2.5 μL of deoxynucleoside triphosphates (dNTPs, 2 mM), 1 U.I. of Taq DNA polymerase (GoTaq G2 Flexi DNA Polymerase M7805; Promega Corp.) and 6.5 μL of H_2_O. PCR was performed in a real‐time PCR (StepOne; Applied Biosystem). The first step of denaturation consisted of 3 min at 94°C, followed by 30 cycles of 30 s at 94°C, 1 min at 57°C, 50 s at 72°C and a final extension of 10 min at 72°C. For the nested PCR step, 1 μL of each first amplification template was added to a new reaction mixture with the same composition as the first. The second denaturation consisted of 3 min at 94°C, followed by 30 cycles of 30 s at 94°C, 1 min at 62°C, 40 s at 72°C and a final extension of 10 min at 72°C. Finally, the second PCR product was displayed with an electrophoretic 2.5% agarose gel. The DNA of *M. pachydermatis* DSM 6172 was employed as a positive control, while the DNA of *M. furfur* ATCC 14521 and *M. globosa* ATCC MYA 4612 was used to confirm the specificity of the amplification process.

### Compounds

Eight compounds were tested: **5g**, **7a** , **7c**, **7k** , **8c**, **10c**, **11b** and **11f**. The chemical structure, formula, molecular weight and inhibition constant (K_I_) of CAs against MP (MpaCA), *M. globosa* (MgCA) and *M. restricta* (MreCA) of the tested compounds are reported in Table 1. The K_i_ is a measure of how strongly an inhibitor binds to an enzyme or receptor. It represents the concentration of the inhibitor needed to reduce the enzyme's activity by 50% compared to the maximum activity (without the inhibitor). The CA kinetic inhibition was evaluated following the method proposed by Khalifah.^23^

**TABLE 1:** Structure, formula, molecular weight (MW) and inhibition data expressed as constant of inhibition (*K*_I_) in the nanomolar range of **5g**, **7a**, **7c**, **7k**, **8c**, **10c**, **11b** and **11f** selenoureido compounds, and the standard sulfonamide inhibitor acetazolamide (AAZ) against *Malassezia pachydermatis* (MpaCA), *M. globosa* (MgCA) and *M. restricta* (MreCA) obtained by means of a stopped‐flow CO_2_ hydrase assay.

Name	Compound structure	Formula	MW (g/mol)	*K*_I_ (nm)^a^		
				MpaCA	MgCA	MreCA
**5g**	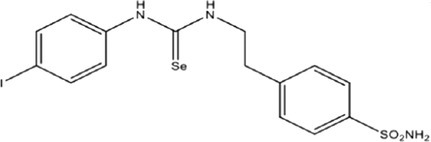	C_15_H_16_IN_3_O_2_SSe	508.24	530.6	689.4	2,701
**7c**	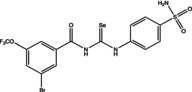	C_15_H_11_BrF_3_N_3_O_4_SSe	545.19	83,470	4,300	6,910
**8c**	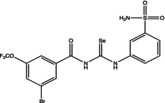	C_15_H_11_BrF_3_N_3_O_4_SSe	545.19	86,110	7,651	7,950
**11b**	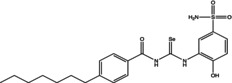	C_21_H_26_N_4_O_5_SSe	496.48	77,970	4,931	8,250
**7a**	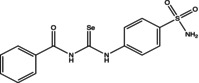	C_14_H_13_N_3_O_3_SSe	382.3	44,770	3,855	4,480
**10c**	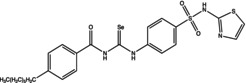	C_24_H_28_N_4_O_3_S_2_Se	563.59	91,880	60,570	5,670
**7k**	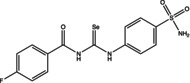	C_14_H_12_FN_3_O_3_SSe	400.29	80,800	8,288	3,800
**11f**	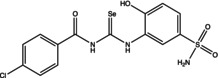	C_14_H_12_ClN_3_O_4_SSe	432.74	54,170	5,131	5,230
AAZ	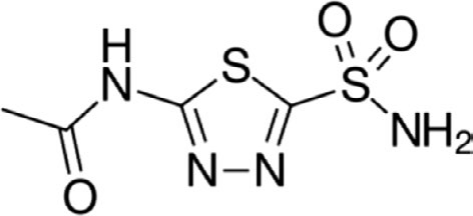	C_4_H_6_N_4_O_3_S_2_	222.25	620.0	74,000	100.0

^a^ Mean from three different assays, by a stopped‐flow technique (errors were in the range of ±5%–10% of the reported values).

### Antifungal activity evaluation

All of the fungal isolates were stored at −80°C in cryovials, then the inoculum preparations for broth microdilution antifungal assays were performed following the CLSI reference methods for antifungal susceptibility testing of yeasts, with some modifications.^24^ Briefly, yeast isolates were inoculated into 6 mL of modified RPMI 1640 broth (Gibco; Life Technologies Ltd) with the addition of ingredients suggested by Rojas et al.^25^ and incubated at 37°C for 48 h.

Antifungal activity also was evaluated on the reference strain of *M. pachydermatis* DSM 6172 (CDC 16334). The fungal inoculum was prepared by suspending four to five colonies of approximately 1 mm diameter in 10 mM phosphate buffer solution (PB, pH 7). The fungal suspension was then adjusted to a final optical density of 0.5 McFarland (1 × 10^6^–5 × 10^6^ colony forming units [CFU/mL]). The tested compounds were dissolved in dimethyl sulfoxide (DMSO) at 25.6 mg/mL, and final concentrations tested ranged from 0.125 to 256 μg/mL. Ketoconazole, MCZ and FCZ (all purchased from Sigma Aldrich) were dissolved in DMSO at a concentration of 25.6 mg/mL and tested at final ranges of 0.008–32 μg/mL for KCZ and 0.125–256 μg/mL for MCZ and FCZ. Growth control with and without 1% DMSO and sterility control were performed. After 48 h of incubation at 37°C, 10 μL of resazurin at 0.02% in PBS (pH 7) was added in each well to assess cell viability and plates were re‐incubated for 24 h at 37°C, before minimum inhibitory concentration (MIC) reading was performed.^26^ A change from blue to pink indicates the reduction of resazurin and therefore fungal growth; the MIC was defined as the lowest drug concentration that prevented this colour change. Quality control isolates (*C. albicans* ATCC 11006 [American Type Culture Collection, Manassas, VA, USA] and *M. pachydermatis* DSM 6172 [German Collection of Microorganisms and Cell Cultures GmbH, DSMZ, Braunschweig, Germany]) were included on each day to check the accuracy of the drug dilutions and the reproducibility of the results. For each test, three experiments were performed, with three replicates each. The MIC value of each tested compound against each field isolate of MP was calculated as the average value of replicates (μg/mL) ± standard deviation (SD).

The CLSI has established MIC breakpoints for azole compounds only for *Candida* spp., while breakpoints for MP have not yet been established, so MIC_50_ and MIC_90_ were considered. MIC_50_ and MIC_90_ indicate the MIC values at which 50% and 90% of the isolates in a test population are inhibited, respectively. Within a series of MICs obtained from test isolates, the MIC_50_ corresponds to the median MIC value, while the MIC_90_ corresponds to the 90th percentile.^27^

### Statistical analysis

The MIC data were subjected to statistical analysis using SPSS (v29). The homogeneity of variance was evaluated using Levene's test. Furthermore, the data were subjected to a normality analysis, with the distribution of the data evaluated using the Shapiro–Wilk test. Based on the results of the homoscedasticity and normality analyses (not shown), which indicated that the data did not follow a normal distribution and that the group variances were not homogeneous, the Kruskal–Wallis test was used to determine whether there were statistically significant differences between the medians of the compared groups. A post hoc analysis was performed to compare tested compounds using a pairwise comparison of mean ranks, with Bonferroni correction. For all analyses carried out, a *p*‐value <0.05 was considered significant.

## RESULTS

Of the 50 samples examined, 36 exhibited a positive result for *Malassezia* growth on SDA. Among the 36 positive samples, 26 were auricular and 10 were cutaneous. Of the positive swabs, seven of the 36 were from patients who had previously received antimicrobial or antifungal therapy for clinical dermatitis or otitis. Of these, five were auricular and two were cutaneous. The previous treatment of the patients and the antifungal active principle used are reported in Table 2.

**TABLE 2:** Isolated *Malassezia pachydermatis* (MP), sampling sites and treatment used on the canine patient before sampling. Where not specified, the treatment was topical.

MP sample	Sampling site	Treatment (molecule)
1	RE	—
2	C	—
3	C	—
4	E	—
5	LE	—
6	E	—
7	E	Miconazole + polymyxin B, florfenicol + terbinafine
8	LE	—
9	E	—
10	C	—
11	E	—
12	E	—
13	LE	—
14	RE	—
15	E	Miconazole + polymyxin B
16	RE	—
17	E	Miconazole
18	E	—
19	RE	—
20	C	—
21	RE	Florfenicol + terbinafine
22	C	—
23	C	—
24	E	—
25	E	Terbinafine
26	C	—
27	RE	—
28	C	Miconazole
29	C	Terbinafine (both topical and per os)
30	LE	—
31	RE	—
32	E	—
33	E	—
34	E	—
35	E	—
36	C	—

Abbreviations: C, cutaneous swab; E, ear canal (not specified); LE, left ear canal; RE, right ear canal.

The electrophoretic profile of the amplicons obtained from all DNA samples by the nested PCR (Figure S1) demonstrated a consistent pattern with the positive control (MP DSM 6172), exhibiting bands at the molecular weight (MW) of 200 bp. The specificity of the nested PCR was confirmed by the observation that neither the DNA of *M. furfur* ATCC 14521 nor that of *M. globosa* ATCC MYA 4612 generated amplification bands.

Antifungal activity of the eight selenoureido compounds was evaluated towards 36 field isolates and MP DSM6172 in addition to the three azole compounds KCZ, MCZ and FCZ. For each compound, MIC_50_ and MIC_90_ (μg/mL) were calculated. Results are reported in Table 3. The solvent of the compounds (1% DMSO) was tested on MP isolates to ensure that it does not exhibit antifungal activity per se, and, as intended, was found to be inactive. The MIC_50_ and MIC_90_ values of all the tested compounds (selenoureido and azoles) on field isolates were higher than the MIC value obtained on the reference MP strain, with the exception of MCZ (for which a value of 16 μg/mL was obtained for both the reference strain and the MIC_50_ of field isolates) and KCZ (for which the MIC_50_ was lower than the MIC of the reference strain: 0.031 vs. 0.016 μg/mL). The lowest MIC_50_ of the selenoureido compounds was found for **7a** (6 μg/mL), followed by **5g** (8 μg/mL); for comparison, among the azoles, KCZ showed the lowest MIC_50_ and MIC_90_ (0.016 and 4 μg/mL, respectively). Compounds **5g**, **7a** and **7k** all showed a lower MIC_50_ than those of MCZ and FCZ. Compound **8c** showed a MIC_50_ value lower than that of FCZ. A similar MIC_90_ value was obtained for all selenoureido compounds and azoles, except for KCZ. Five of 36 isolates showed a MIC > MIC_90_ to at least one azole compound. Three of these isolates showed a MIC > MIC_90_ to KCZ, one to MCZ and one to FCZ. Thirteen of 36 isolates showed a MIC_50_ < MIC sample ≤ MIC_90_ to KCZ, 11 of 36 to MCZ and 17 of 36 to FCZ. All of the isolates with a MIC > MIC_90_ were from ear swabs from patients with OE (five of 26) and none of these had recently been treated with azoles (only one was treated with florfenicol + terbinafine). All of the selenoureido compounds showed at least one MIC value lower than azoles on MP field isolates with MIC > MIC_90_, except for **7c** and **11b**. Compound **7a** showed the best activity on these five isolates, with MIC values lower than those for azoles on four of five isolates, followed by **10c** and **8c** (three of five each), **7k** (two of five) and **11f** and **5g** (one of five each).

**TABLE 3:** Minimum inhibitory concentration (MIC) values (mean MIC μg/mL ± SD) of **5g**, **7a**, **7c**, **7k**, **8c**, **10c**, **11b** and **11f** selenoureido compounds, ketoconazole (KCZ), miconazole (MCZ) and fluconazole (FCZ) obtained on all considered field isolates.

Isolates	Compounds										
	11f	10c	7k	8c	7c	5g	11b	7a	KCZ	MCZ	FCZ
MP DSM6172	0.5 ± 0	0.75 ± 0.35	0.5 ± 0	2 ± 0	0.5 ± 0	1 ± 0	8 ± 0	0.5 ± 0	0.031 ± 0	16 ± 0	8 ± 0
1	6 ± 2.83	6 ± 2.83	8 ± 1.41	3 ± 1.41	4 ± 0	3 ± 1.41	1.5 ± 0.71	2 ± 0	0.031 ± 0	8 ± 0	12 ± 5.66
2	128 ± 0	64 ± 0	8 ± 0	16 ± 0	24 ± 11.31	4 ± 0	128 ± 0	6 ± 2.83	0.031 ± 0	32 ± 0	32 ± 0
3	128 ± 0	64 ± 0	32 ± 0	64 ± 0	24 ± 11.31	24 ± 11.31	128 ± 0	32 ± 0	0.031 ± 0	12 ± 5.66	16 ± 0
4	128 ± 0	2 ± 0	4 ± 0	8 ± 0	24 ± 11.31	4 ± 0	128 ± 0	2 ± 0	0.031 ± 0	8 ± 0	8 ± 0
5	128 ± 0	128 ± 0	24 ± 11.31	2 ± 0	4 ± 0	4 ± 0	128 ± 0	24 ± 11.31	0.094 ± 0.04	8 ± 0	8 ± 0
6	32 ± 0	32 ± 0	32 ± 0	8 ± 0	24 ± 11.31	4 ± 0	64 ± 0	2 ± 0	0.031 ± 0	12 ± 5.66	8 ± 0
7	128 ± 0	16 ± 0	6 ± 2.83	16 ± 0	24 ± 11.31	3 ± 1.41	128 ± 0	2 ± 0	0.063 ± 0	16 ± 0	16 ± 0
8	128 ± 0	32 ± 0	128 ± 0	32 ± 0	16 ± 0	4 ± 0	128 ± 0	32 ± 0	0.125 ± 0	8 ± 0	8 ± 0
9	128 ± 0	32 ± 0	3 ± 1.41	32 ± 0	64 ± 0	4 ± 0	128 ± 0	4 ± 0	0.125 ± 0	16 ± 0	32 ± 0
10	128 ± 0	32 ± 0	4 ± 0	64 ± 0	64 ± 0	4 ± 0	128 ± 0	6 ± 2.83	0.016 ± 0	32 ± 0	32 ± 0
11	24 ± 11.31	24 ± 11.31	64 ± 0	32 ± 0	8 ± 0	6 ± 2.83	2 ± 0	6 ± 2.83	0.094 ± 0.044	8 ± 0	8 ± 0
12	64 ± 0	64 ± 0	64 ± 0	128 ± 0	32 ± 0	8 ± 0	4 ± 0	64 ± 0	0.063 ± 0	4 ± 0	4 ± 0
13	128 ± 0	128 ± 0	16 ± 0	64 ± 0	128 ± 0	8 ± 0	128 ± 0	6 ± 2.83	0.016 ± 0	64 ± 0	64 ± 0
14	128 ± 0	128 ± 0	8 ± 0	128 ± 0	32 ± 0	6 ± 2.83	128 ± 0	4 ± 0	0.016 ± 0	16 ± 0	32 ± 0
15	128 ± 0	128 ± 0	64 ± 0	128 ± 0	16 ± 0	8 ± 0	128 ± 0	64 ± 0	0.016 ± 0	4 ± 0	4 ± 0
16	128 ± 0	128 ± 0	128 ± 0	8 ± 0	16 ± 0	16 ± 0	128 ± 0	8 ± 0	16 ± 0	64 ± 0	64 ± 0
17	32 ± 0	4 ± 0	1 ± 0	1 ± 0	32 ± 0	8 ± 0	128 ± 0	64 ± 0	0.031 ± 0	32 ± 0	64 ± 0
18	16 ± 0	16 ± 0	3 ± 1.41	32 ± 0	64 ± 0	6 ± 2.83	128 ± 0	6 ± 2.83	0.016 ± 0	32 ± 0	32 ± 0
19	32 ± 0	2 ± 0	128 ± 0	16 ± 0	12 ± 5.66	32 ± 0	96 ± 45.25	32 ± 0	8 ± 0	8 ± 0	12 ± 5.66
20	2 ± 0	4 ± 0	2 ± 0	8 ± 0	32 ± 0	4 ± 0	128 ± 0	2 ± 0	0.016 ± 0	16 ± 0	32 ± 0
21	4 ± 0	6 ± 2.83	3 ± 1.41	12 ± 5.66	32 ± 0	4 ± 0	128 ± 0	4 ± 0	0.016 ± 0	64 ± 0	128 ± 0
22	128 ± 0	64 ± 0	32 ± 0	32 ± 0	16 ± 0	8 ± 0	128 ± 0	64 ± 0	0.016 ± 0	4 ± 0	64 ± 0
23	128 ± 0	128 ± 0	4 ± 0	6 ± 2.83	32 ± 0	12 ± 5.66	8 ± 0	24 ± 11.31	0.016 ± 0	8 ± 0	12 ± 5.66
24	128 ± 0	32 ± 0	64 ± 0	8 ± 0	128 ± 0	4 ± 0	3 ± 1.41	32 ± 0	0.016 ± 0	12 ± 5.66	12 ± 5.66
25	128 ± 0	4 ± 0	128 ± 0	8 ± 0	16 ± 0	32 ± 0	2.5 ± 0.71	128 ± 0	0.016 ± 0	16 ± 0	16 ± 0
26	3 ± 1.41	8 ± 0	1.5 ± 0.71	6 ± 2.83	32 ± 0	6 ± 2.83	3 ± 1.41	0.38 ± 0.18	0.016 ± 0	8 ± 0	16 ± 0
27	64 ± 0	24 ± 11.31	8 ± 0	16 ± 0	12 ± 5.66	16 ± 0	128 ± 0	16 ± 0	0.016 ± 0	8 ± 0	8 ± 0
28	64 ± 0	16 ± 0	4 ± 0	8 ± 0	32 ± 0	24 ± 11.31	4 ± 0	128 ± 0	0.016 ± 0	8 ± 0	8 ± 0
29	128 ± 0	32 ± 0	32 ± 0	64 ± 0	32 ± 0	16 ± 0	4 ± 0	64 ± 0	0.016 ± 0	8 ± 0	8 ± 0
30	128 ± 0	6 ± 2.83	2 ± 0	4 ± 0	12 ± 5.66	12 ± 5.66	128 ± 0	1 ± 0	0.016 ± 0	64 ± 0	64 ± 0
31	128 ± 0	6 ± 2.83	6 ± 2.83	16 ± 0	32 ± 0	32 ± 0	64 ± 0	2 ± 0	0.016 ± 0	128 ± 0	32 ± 0
32	128 ± 0	64 ± 0	32 ± 0	12 ± 5.66	8 ± 0	128 ± 0	128 ± 0	3 ± 1.41	4 ± 0	16 ± 0	32 ± 0
33	128 ± 0	128 ± 0	32 ± 0	64 ± 0	64 ± 0	128 ± 0	128 ± 0	12 ± 5.66	0.016 ± 0	64 ± 0	64 ± 0
34	128 ± 0	128 ± 0	64 ± 0	128 ± 0	64 ± 0	128 ± 0	128 ± 0	32 ± 0	0.016 ± 0	16 ± 0	32 ± 0
35	128 ± 0	128 ± 0	16 ± 0	32 ± 0	64 ± 0	128 ± 0	128 ± 0	4 ± 0	8 ± 0	64 ± 0	64 ± 0
36	128 ± 0	128 ± 0	4 ± 0	128 ± 0	12 ± 5.66	8 ± 0	2 ± 0	4 ± 0	0.016 ± 0	32 ± 0	64 ± 0
MIC50	128	32	12	16	28	8	128	6	0.016	16	24
MIC90	128	128	128	128	64	128	128	64	4	64	64

**FIGURE 1:**
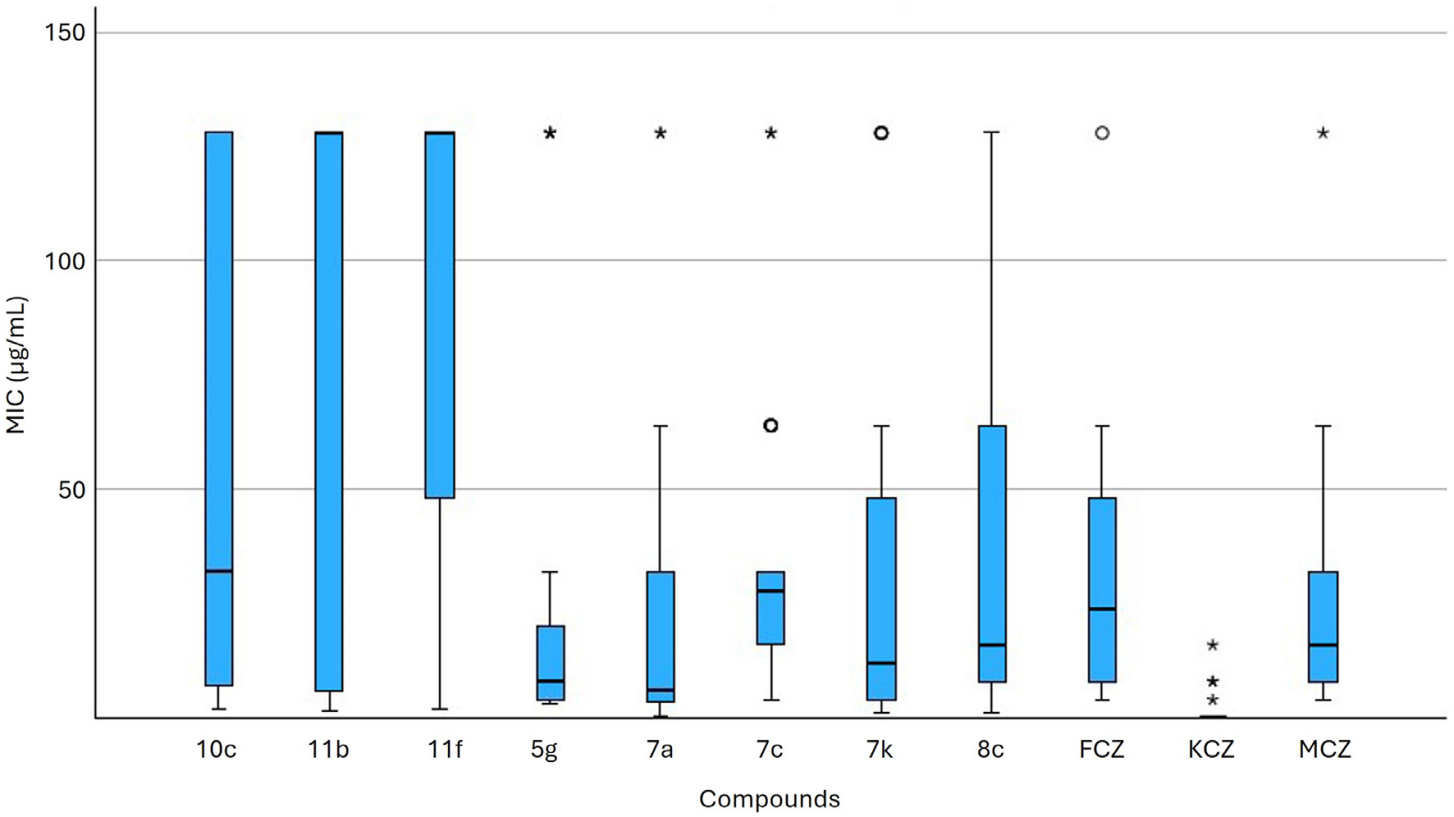
Box‐plot graph of the minimum inhibitory concentration (MIC) values of the tested compounds. Tested compounds are shown on the *x*‐axis, and MIC values (μg/mL) of each compound against *Malassezia pachydermatis* field isolates are shown on the *y*‐axis. FCZ, fluconazole; KCZ, ketoconazole; MCZ, miconazole; *, extreme outlier;°, outlier.

Figure 1 shows a boxplot of the MIC values of the tested compounds and reference antifungal drugs. The MIC_50_ values of the reference drugs FCZ, MCZ, and KCZ were 24.0, 16.0 and 0.016 μg/mL, respectively. Four of the tested compounds, namely **7a**, **5g**, **8c** and **7k**, exhibited comparable MIC_50_ values to those of FCZ and MCZ, with values of 6.0, 8.0, 16.0 and 12.0 μg/mL, respectively. However, a greater dispersion of the interquartile ranges around the median values was observed within selenoureido compounds in comparison to the azoles.

The Kruskal–Wallis test, conducted with 10 degrees of freedom, yielded a test statistic value of 142.022 (*p* < 0.001), indicating a significant difference in MIC values among the compounds under comparison. Pairwise comparisons between the selenoureido compounds and the reference azoles showed that the MIC values of KCZ were significantly lower than those of all the other compounds tested. However, compounds **5g**, **7a**, **7k** and **8c** showed lower MIC_50_ values compared to those of MCZ and FCZ. Statistical analysis revealed a significant difference between compound **5g** (*p* = 0.005) and compound **7a** (*p* = 0.005) compared to FCZ. No significant difference was observed for compound **7k** compared to FCZ (*p* = 0.076), and for compounds **5g**, **7a** and **7k** compared to MCZ (*p* = 0.054, *p* = 0.055 and *p* = 0.361, respectively). Finally, compound **8c** displayed substantial variability in MIC values, which may affect the accuracy of its MIC_50_ estimation and resulted in no significant difference compared to the reference azoles FCZ (*p* = 0.642) and MCZ (*p* = 0.69).

## DISCUSSION

In recent years, the diagnosis of *M. pachydermatis* skin infection in domestic animals has increased, owing to the more accurate identification provided by molecular analyses and the recognition of the pathogenic role of the yeast in relation to predisposing factors. Therefore, the need for treatments, in particular those for topical application, has increased and the most used molecules are represented by azoles. Although the onset of resistance to antifungals is slower than to antibacterials, several therapeutic failures have been reported in both animals and humans as a result of phenomenon.^2^, ^28^, ^29^, ^30^

The present study investigated the efficacy of eight novel selenoureido antifungal compounds on a collection of 36 MP field isolates isolated from ear and cutaneous swabs in dogs with dermatitis and OE, a population representative of the heterogeneity commonly encountered in clinical practice.

As reported in a previous work, tested selenoureido compounds have a dual mode of action, combining the toxicity of selenium with activity on yeast carbonic anhydrases, which have already shown great efficacy against different *Malassezia* reference isolates.^17^ Moreover, the selenoureido compounds tested demonstrated a good tolerability when tested on human keratinocyte cell line, suggesting that topical administration could be a valid route.^17^

The data obtained from the evaluation of antifungal activity on the clinical isolates showed that, despite the wide variability observed among MIC values for selenoureido compounds, some compounds (**5g**, **7a** and **7k**) have MIC_50_ values lower than those of MCZ and FCZ.

Given the crucial role of yeast CAs in key metabolic processes essential for yeast survival, the inhibition of these enzymes, as indicated by the kinetic inhibition data in Table 1, are likely to have contributed to the observed growth inhibition. This inhibition could be particularly valuable in isolates with high MIC values for azole drugs. Specifically, **5g**, which is the compound with the most effective antifungal activity, demonstrated a potent inhibition constant against MpaCA in the low micromolar range (*K*_I_ = 0.53 μm). By contrast, **7a** and **7k**, which showed low MIC_50_ values, exhibited inhibition constants in the medium/high micromolar range (*K*_I_ = 44.7 and 80 μm, respectively), which are indicative of a strong connection with the target. In particular, compounds **5g** and **7a** showed, from the boxplot graph of the medians, a lower variability in MIC results than for MCZ and FCZ, and a statistical difference from those of FCZ, configuring themselves as good candidates for further investigations.

However, KCZ was the most effective antifungal, as stated by other authors^31^, ^32^ and also the one to which a higher number of isolates were above the 90th percentile of MIC values (three of 36). In particular, Watanabe et al. obtained MIC_90_ values for KCZ in MP isolates obtained from affected dogs with atopic dermatitis similar to those presented in this study (4.21 μg/mL in Taiwan and 5.2 μg/mL in Japan).^33^ These findings were also supported by Cafarchia et al., who detected higher mean MIC values to KCZ in MP isolates from patients with skin lesions than those from healthy dogs, reflecting a possible influence of the prolonged exposure to azole antifungals inducing resistance.^34^ In any case, the absence of commonly accepted recommendations for interpretation of *Malassezia* susceptibility to antifungals constitutes an obstacle to the clear interpretation of azole MIC results.

A possible explanation for the wide variability of MIC results obtained with the selenoureido compounds may be diversity in genetic types among different MP isolates, as highlighted by Aizawa et al.^35^ Further study should investigate whether the genetic diversity among MP isolates could affect the antifungal activity of selenoureido compounds, as well as evaluate which resistance mechanisms are involved in azole resistance in the five resistant isolates.

Moreover, in vivo experiments should be carried out to evaluate the effectiveness and safety of topical treatment with selenoureido compounds in MP infections in dogs.

## CONCLUSIONS

This study demonstrated a good efficacy of the eight tested selenoureido compounds, notably compounds **5g** and **7a**, as potential novel antifungal compounds against canine clinical isolates of *M. pachydermatis*. The evaluation of antifungal activity on clinical samples allowed the compounds to be configured as potentially effective antimycotics in the topical treatment of skin and ear infections in dogs, comparable to the most commonly used azoles, such as MCZ and FCZ. Furthermore, as reported in our previous work, selenoureido compounds exhibit selective activity against MP, thereby preserving the integrity of the remaining mycobiome. From a ‘One Health’ perspective, their selectivity of action could encourage the use of selenoureido compounds for the treatment of MP in animals, allowing a reduction in the use of azole compounds and thereby counteracting the development of azole resistance.

## AUTHOR CONTRIBUTIONS

**Costanza Spadini:** Conceptualization; investigation; writing – original draft; methodology; writing – review and editing; data curation; formal analysis. **Nicolò Mezzasalma:** Conceptualization; investigation; writing – original draft; writing – review and editing; software; formal analysis. **Amienwanlen Eugene Odigie:** Software; data curation; writing – review and editing. **Andrea Angeli:** Conceptualization; investigation; formal analysis; writing – review and editing; methodology. **Fabrizio Carta:** Conceptualization; investigation; writing – review and editing; data curation; supervision. **Silvia Selleri:** Conceptualization; data curation; supervision; writing – review and editing. **Emanuele Gandolfo:** Investigation; writing – review and editing. **Simone Taddei:** Writing – review and editing; writing – original draft; software; supervision. **Valentina Franceschi:** Investigation; formal analysis; writing – review and editing; data curation. **Sergio Minesso:** Investigation; writing – review and editing; formal analysis; data curation. **Claudiu T. Supuran:** Conceptualization; supervision; data curation; writing – review and editing; validation; resources. **Clotilde Silvia Cabassi:** Resources; supervision; conceptualization; writing – original draft; writing – review and editing; validation; project administration.

## FUNDING INFORMATION

Self‐funded.

## CONFLICT OF INTEREST STATEMENT

F.C., A.A., S.S., C.T.S., C.S.C. and C.S. declare themselves as inventors of the patent for industrial invention WO 2023/073634 A1, entitled ‘Carbamoselenoyl derivatives as anti‐infective agents’, published 4 May 2023.

## Supporting information


Figure S1.

## Data Availability

The data that support the findings of this study are available from the corresponding author upon reasonable request.
