# Supplementary material for: Comparative efficacy of selenoureido carbonic anhydrase inhibitors and azole antifungal drugs against clinical isolates of Malassezia pachydermatis
Source: Vet Dermatol. 2025 Mar 16;36(3):302–13. doi: 10.1111/vde.13336 (PMC12058572; doi:10.1111/vde.13336)
Supplement: Supplementary file 1 — Figure S1. [file VDE-36-302-s001.docx]

**Supplementary Materials**

**New pharmaceutical proposal for Malassezia pachydermatis clinical isolates in dogs: selenoureido carbonic anhydrase inhibitor compounds compared to azoles**

**FIGURE S1.** **Agar gel electrophoresis of nested PCR products targeting the internal transcribed spacer (ITS) region of Malassezia pachydermatis.**

The amplicon patterns of the 36 field isolates, all displaying the same profile as MP DSM6172 with a 220 bp amplicon, are shown.

**
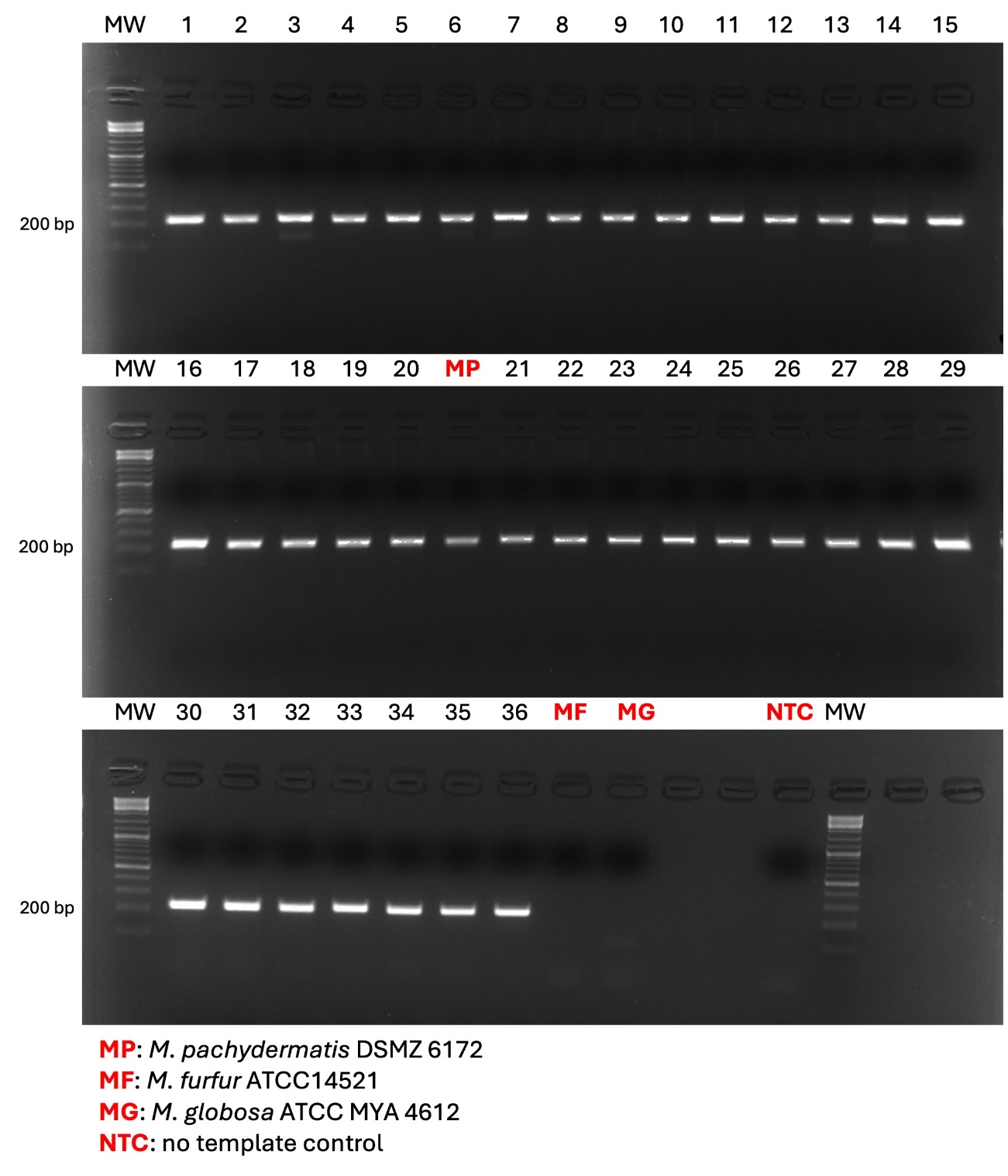
**
